# Empathy and Love as Protective Factors in Opioid Use Disorder Recovery

**DOI:** 10.1080/02791072.2026.2710067

**Published:** 2026-08-07

**Authors:** Kobe R. Hanson, Nina A. Cooperman, Eric L. Garland

**Affiliations:** a Department of Psychiatry, School of Medicine, University of California San Diego, San Diego, CA, USA; b Division of Addiction Psychiatry, Robert Wood Johnson Medical School, Rutgers University, Piscataway, NJ, USA; c Sanford Institute for Empathy and Compassion, University of California San Diego, San Diego, CA, USA

**Keywords:** Love and empathy, methadone treatment, opioid use disorder (OUD), prosocial emotions, social attachment theory

## Abstract

Opioid use disorder (OUD) is a debilitating condition that impacts multiple domains of life, including social attachment systems, which makes recovery and relapse prevention challenging. The *Brain Opioid Theory of Social Attachment* suggests opioids hijack mechanisms involved in love and bonding. Previous studies show that social interaction can help reduce opioid use in both animals and humans, but little is known about whether prosocial emotions like love and empathy can protect against opioid use among people treated with medications for OUD. In this cross-sectional study, data were analyzed from 189 adults receiving methadone treatment for OUD at community-based programs in New Jersey and Utah. Surveys measured opioid use, empathy, love, along with covariates including pain, anxiety, depression, social support, and stigma. Participants reported lower levels of love and empathy compared to normative samples. In zero-inflated Poisson, higher love and empathy scores were significantly associated with fewer episodes of opioid use, even after adjusting for covariates. Anxiety, pain, and social support were significant predictors, with social support unexpectedly linked to greater opioid use. Findings suggest that love and empathy can buffer against opioid use, consistent with theories that support the use of prosocial emotions that target the same pathways as opioids.

## Introduction

The opioid epidemic continues to impact communities as addiction and overdose rates steadily escalate. Between 2019 and 2023, opioid-related overdoses increased from over 49,000 to approximately 79,000, underscoring the severity of the crisis (Garnett and Miniño 2024). Standard treatments for opioid use disorder (OUD), such as medications for OUD (e.g., methadone, buprenorphine) and counseling, aim to address the physiological and behavioral dimensions of addiction. Yet, emerging research suggests that social attachment (e.g., love, empathy) may play a vital role in OUD recovery and relapse prevention.

Beyond their adverse health-related consequences, chronic substance use and behavioral addictions have been associated with disruptions in social attachment and interpersonal functioning. Substances such as alcohol, methamphetamine, cocaine, and opioids, as well as behavioral addictions (e.g., gambling), have been linked to increased social isolation, altered reward processing, and reductions in empathic capacity (Heilig et al. 2016; Volkow, Koob, and McLellan 2016). Although these conditions differ in pharmacology and behavioral expression, they engage overlapping reward, stress, and social-affective systems and may promote reward substitution processes that diminish social connection over time.

Opioid use represents one example of these processes. Chronic opioid use, in particular, may impact social attachment by disrupting social bonding mechanisms. The Brain Opioid Theory of Social Attachment posits that the endogenous opioid system is fundamental for forming and maintaining relationships (Machin and Dunbar 2011; Panksepp et al. 1978). For example, physical contact between mother and infant triggers endogenous opioid release, producing comfort and reducing distress – mechanisms believed to underlie the formation of strong attachment bonds (Machin and Dunbar 2011; Nelson and Panksepp 1998). Chronic and high-dose opioid use downregulates the endogenous opioid system, rendering individuals increasingly dependent on exogenous opioids to maintain a sense of well-being (Higginbotham et al. 2022). Through this neurobiological process, opioid-induced euphoria supersedes the reward obtained from interpersonal relationships. Moreover, opioid use is theorized to dysregulate social homeostatic mechanisms in the brain that regulates the need for social connection, hypothetically reinforcing continued opioid use as a means of coping with consequent feelings of loneliness (Løseth, Eikemo, and Leknes 2025). In support of this theory, opioid receptor blockade has been shown to weaken perception of social connection, further linking opioid use to impairments in attachment (Inagaki et al. 2016). However, this theory has not been tested in the context of addictions treatment.

Since opioid use fosters isolation and disrupts social connectivity, processes linked with social attachment, like love and empathy, may serve as protective factors against addictive behavior. Preclinical evidence supports the idea that experiences of love and attachment engage the endogenous opioid system. For example, in research on prairie voles, a species that forms long-term monogamous bonds, activating opioid receptors is important for forming adult social attachments (Burkett et al. 2011). When these receptors are blocked, voles fail to develop partner preference, despite previous mating and close social contact. This finding suggests that endogenous opioids mediate the rewarding aspects of social bonding, offering a biological explanation why emotionally rich relationships might buffer against opioid misuse.

Social connection also appears to counteract the drive for opioid consumption directly. In one study, rats chose social interaction over heroin in more than 80% of trials, even after developing addiction-like behavior, and the effect persisted across different heroin doses (Venniro et al. 2018). Similarly, isolated rats consumed more morphine than socially housed rats, suggesting that the absence of social contact may heighten vulnerability to opioid intake (Raz and Berger 2010). Further, this effect was fully reversed with just 60 minutes of daily social interaction, indicating that even brief, consistent social contact may restore reward balance and reduce drug-seeking behavior. Although some of these effects may reflect limited access to alternative rewarding behaviors or stimuli rather than social attachment per se, preclinical evidence nevertheless suggests that social interaction functions as a powerful alternative reinforcer.

Translating these findings to humans, research shows that social engagement plays a similarly protective role in addiction recovery. For instance, strong family and peer support systems encourage individuals with OUD to seek and remain in treatment (Hogue et al. 2021). Also, higher social support and family cohesion are associated with future drug use and recovery outcomes (Simpson et al. 2000; Warren, Stein, and Grella 2007). These findings suggest that emotionally supportive relationships can promote sustained recovery.

However, to what extent emotional states associated with social attachment (e.g., love, empathy) are associated with improved addiction treatment outcomes remains unknown. Given that prosocial emotions activate the same reward pathways as opioids, feelings of love and empathy might serve as a protective factor for people in recovery. This research aims to test the hypothesis that feelings of love and empathy will be inversely associated with opioid use among people in addiction recovery. The study seeks to address a gap in addiction studies about the role played by prosocial emotions in OUD recovery.

## Methods

This cross-sectional study examined data from a sample of participants (*N* = 189) participating in clinical trials on interventions for patients with OUD receiving methadone treatment in community-based opioid treatment programs (OTPs). The present study utilized baseline survey data collected after the study enrollment but before randomization; the RCT outcome results will be presented elsewhere. Participants were recruited from one of several OTPs in New Jersey and Utah. Inclusion criteria were: 1) English-fluent adults at least 18 years old; and 2) currently receiving methadone treatment for OUD. Exclusion criteria: 1) severe cognitive impairment or psychosis; 2) high suicide risk; and 3) inability to participate in study procedures.

Following an initial screening, eligible, interested adults completed informed consent procedures. Next, participants completed baseline assessments, which included several questionnaires assessing demographic and clinical information. All participants were remunerated for their participation. The study protocol was approved by the Rutgers Institutional Review Board.

### Measures

#### Dependent variable

##### Opioid use.

Participants completed a question from the validated Addiction Severity Index (McLellan et al. 1992) assessing the number of episodes of opioid use in the past 30 days. Participants were asked about use of heroin, fentanyl, and other opioids (e.g., oxycodone, hydrocodone), with the total number of instances of use summed across these various types of opioids.

#### Independent variables

##### Empathy.

Empathy was assessed with the self-reported abbreviated 8-item version of the Empathy Quotient on a 4-point Likert scale (1 = strongly disagree to 4 = strongly agree) (P. Loewen, Lyle, and Nachshen 2009). This includes statements such as “I find it easy to put myself in somebody else’s shoes,” and “Other people often say that I am insensitive, though I don’t always see why.” Four items were positively worded and four were reverse scored. Total scores were computed by summing responses, with higher scores indicating greater empathic capacity. The EQ-8 has demonstrated good internal consistency (α = .76) and construct validity in prior research and is derived from the widely used Empathy Quotient (Baron-Cohen and Wheelwright 2004).

##### Love.

Love was assessed using the 6-item love subscale of the Dispositional Positive Emotion Scale (Shiota, Keltner, and John 2006). Participants rated their agreement with each item on a 7-point Likert scale ranging from 1 (strongly disagree) to 7 (strongly agree). Items reflect a disposition toward feelings of trust, closeness, and warmth in relationships (e.g., “I develop strong feelings of closeness to people easily”). Scores were calculated by averaging responses across items, with higher scores indicating a greater dispositional tendency to experience love. The love subscale has demonstrated good internal consistency in prior research.

#### Covariates

##### Pain.

Pain was assessed with a single item from the PROMIS assessing average pain in the past week on a 0 to 10 numeric rating scale which has demonstrated strong convergent validity with multi-item pain measures in clinical research (Amtmann et al. 2010).

##### Anxiety.

Anxiety symptoms were assessed using the Generalized Anxiety Disorder 2-item scale (Spitzer et al. 2006), a brief self-report measure evaluating the frequency of core anxiety symptoms over the past two weeks. Participants responded to two items, “Feeling nervous, anxious, or on edge” and “Not being able to stop or control worrying,” using a 4-point Likert scale ranging from 0 (not at all) to 3 (nearly every day). Total scores ranged from 0 to 6, with higher scores reflecting greater anxiety severity. The GAD-2 has demonstrated strong sensitivity and specificity for detecting generalized anxiety in both clinical and general populations (Kroenke et al. 2007).

##### Depression.

Depression symptoms were assessed using the 3-item Patient Health Questionnaire (Perlis et al. 2025), a brief self-report measure derived from the PHQ-9 (Kroenke, Spitzer, and Williams 2001). The PHQ-3 includes three core items: (1) little interest or pleasure in doing things, (2) feeling down, depressed, or hopeless, and (3) feeling bad about yourself or like a failure. Participants rated how often they experienced each symptom over the past two weeks on a 4-point Likert scale ranging from 0 (not at all) to 3 (nearly every day). Total scores were calculated by summing the three items (range: 0 to 9), with higher scores indicating greater depression severity. The PHQ-3 has demonstrated good internal consistency in prior research (α = .85).

##### Social support.

Social support was assessed using the Multidimensional Scale of Perceived Social Support (MSPSS; Zimet et al. 1988), a 12-item self-report measure that evaluates perceived support from three sources: family, friends, and a significant other. Participants responded to items using a 7-point Likert-type scale ranging from 1 (very strongly disagree) to 7 (very strongly agree). Example items include “My family really tries to help me” and “I can count on my friends when things go wrong.” Subscale scores were calculated by averaging the four relevant items, and a total score was computed by averaging across all 12 items. The MSPSS has demonstrated strong internal consistency, with Cronbach’s alpha coefficients of .91 for the Significant Other subscale, .87 for Family, and .85 for Friends.

##### Substance use stigma.

Substance use stigma was assessed using the Substance Use Stigma Mechanism Scale (SUSMS; Smith et al. 2016), an 18-item self-report measure grounded in Link and Phelan’s stigma framework. The SUSMS evaluates three mechanisms of stigma, enacted stigma (experiences of discrimination), anticipated stigma (expectations of future discrimination), and internalized stigma (self-directed shame and devaluation), across two sources, family members and healthcare providers. Items are rated on 5-point Likert-type scales, with anchors ranging from 1 (never, very unlikely, or strongly disagree) to 5 (very often, very likely, or strongly agree), depending on the subscale. Subscale scores were computed as the mean of relevant items, with higher scores indicating greater perceived stigma. In the original validation study, the SUSMS demonstrated excellent internal consistency (Cronbach’s α = .90–.93 across subscales).

### Statistical analysis

All analyses were conducted using SPSS version 29.0.2. We first calculated descriptive statistics to discern the zero-order relationships among love, empathy, and opioid use. As a descriptive analysis, one sample t-tests were used to compare our sample to the normed average love (Dixson, Anderson, and Keltner 2018) and empathy ratings in samples of people without substance use disorders. Because opioid use was highly zero-inflated in our sample, we employed a zero inflated Poisson (ZIP) regression analysis for all subsequent analytic steps. ZIP models can account for the excessive number of zeros common in substance use data (Atkins et al., 2013).

We computed a ZIP model to test whether love and empathy scores explained a statistically significant portion of variance in episodes of opioid use, after controlling for a number of theoretically-informed confounding variables (pain, anxiety, depression, social support, and substance use stigma) as covariates. The ZIP model simultaneously estimates two components: one for excess zeros using a logit approach and another for the count data using a Poisson distribution.

## Results

### Participant characteristics

Sample characteristics are reported in Table 1. Participants were, on average, 46.5 years old (±13.3), and 46% were women. Sixty-two percent were White, 28% were Black, and 10% identified as American Indian/Alaska Native, Asian, or another race. Forty percent had never been married, 17% were divorced, and 7% were widowed. Over half (55%) reported annual incomes below $10,000, and 21% had not completed high school. In the past 30 days, 41% reported opioid use, and participants had been receiving methadone treatment for an average of 4.85 years (±6.03). At baseline, participants reported clinically significant symptoms of depression (*M* = 2.45 ± 2.19) and anxiety (*M* = 2.59 ± 1.98).

In our sample, love (DPES) scores were significantly lower than the norm mean DPES score (*M* = 4.65) reported for individuals (*N* = 921) without substance use disorder diagnoses (Dixson, Anderson, and Keltner 2018), *t* = −7.73, *p* < .001, Cohen’s D = 0.56. Empathy (EQ-8) scores in our sample were significantly lower than the norm mean EQ-8 score (*M* = 9.60) reported for individuals (*N* = 4682) without substance use disorder diagnoses (Loewen, Lyle, and Nachshen 2009), *t* = −22.32, *p* < .001, Cohen’s D = 1.61.

Table 2 presents the ZIP model examining empathy and love predicting the number of opioid use days in the past 30 days (*N* = 189). On average, participants reported using opioids approximately 22 of the past 30 days.

### Love and empathy as predictors of opioid use

ZIP model results are depicted in Table 2. In the count portion of the ZIP model, higher levels of love (B = −0.068, SE = 0.02, *p* < .001) and empathy (B = −.043, SE = 0.011, *p* < .001) were significantly associated with fewer episodes of opioid use (Figure 1). Neither love nor empathy were significant predictors in the zero-inflated portion of the model. Other covariates were also significant predictors in the count portion of the model, including anxiety, and social support, but no covariate was a significant predictor in the zero-inflated portion of the model. Pain was inversely associated with opioid use.

To clarify this counterintuitive finding, we computed exploratory moderation analyses to test love/empathy as moderators of the effect of pain on opioid use. The interaction effect of love and pain (B = 0.050, SE = 0.13, *p* < .001) and empathy and pain (B = 0.037, SE = 0.01, *p* < .001) on episodes of opioid use were both significant for the count portion of the zero-inflated models but not for the zero-inflated portion of the models. Both models showed the same pattern: for patients with lower levels of love/empathy, pain was strongly negatively associated with days of opioid use. In contrast, for patients with higher levels of love/empathy, pain was less strongly negatively associated with opioid use.

## Discussion

Here we found that higher levels of love and empathy predicted less opioid use among a sample of people with OUD in community-based addictions treatment. Love and empathy remained significant predictors even after controlling for a wide array of confounding variables, suggesting that beyond being mere proxies for low levels of depression and anxiety, or high levels of social support, love and empathy confer unique protective effects on drug use.

These findings align with the Brain Opioid Theory of Social Attachment (Machin and Dunbar 2011; Panksepp et al. 1978), which posits that the brain’s endogenous opioid system plays a critical role in sustaining social relationships by reinforcing the pleasure and comfort of emotional closeness. Consistent with this view, endogenous opioid signaling has also been implicated in other addictions, including both substance and behavioral addictions, where opioidergic mechanisms contribute to the rewarding and pleasurable effects of addictive behaviors (Clark, Boileau, and Zack 2019; Reed, Butelman, and Kreek 2017). Importantly, endogenous opioid system functioning under chronic methadone treatment is not well understood, and it remains unclear to what extent methadone normalizes endogenous opioid signaling compared to other opioids such as heroin or fentanyl. That said, it is possible that socially affiliative emotional experiences might modulate the endogenous opioid system despite potential opioidergic receptor downregulation due to chronic methadone exposure. Taken together, these findings suggest that the capacity for prosocial feelings may remain intact despite opioid-system dysregulation under methadone treatment.

If feelings of love and empathy activate the same opioidergic pathways that heroin or fentanyl target, then individuals with greater access to these natural sources of reward may have less need to rely on exogenous opioids as a source of well-being. Conversely, chronic recreational opioid use may hijack these same circuits, thereby diminishing the reward value of interpersonal connection. From this hedonic homeostatic perspective (Koob and Le Moal 1997), fostering love and empathy could serve as a reward substitution, replacing drug-derived reinforcement for the intrinsic satisfaction of meaningful social bonds. A shift of reward value from opioids to social connection could help reduce use and promote sustained recovery. This notion is consistent with preclinical work demonstrating that fostering social bonding in rats reduces opioid consumption and conditioned responses (Raz and Berger 2010; Venniro et al. 2018). Alternatively, love and empathy may influence opioid use through non-opioidergic mechanisms. Oxytocin and serotonin represent additional candidate neuromolecular systems that may mediate this association (Lyubomirsky 2022; Sarnyai and Kovács 2014).

Moreover, love and empathy may buffer opioid use by preserving the social-emotional capacities that chronic drug use often erodes. Chronic opioid exposure appears to degrade the very skills needed to form and sustain interpersonal bonds. For example, individuals receiving buprenorphine/naloxone treatment performed significantly worse than healthy controls on facial emotion recognition and theory of mind tasks, skills essential for accurate social perception (Yazgeç et al. 2023). Similarly, Terrett et al. (2020) reported that long-term opioid users show broad impairments in empathy, including reduced accuracy in identifying basic emotions, diminished performance on theory of mind tasks, and an absence of facial mimicry (an automatic marker of affective resonance). These impairments mirror broader patterns in which reduced social-emotional functioning is linked to greater opioid misuse risk, as seen in older adults where social withdrawal independently predicts prescription opioid misuse (Lam, Vuolo, and Kelly 2025). Together, these findings suggest that prolonged opioid use can push individuals into a downward spiral: as social-emotional functioning declines, it becomes harder to form the very relationships that could provide love and empathy, further entrenching reliance on drugs as a source of pleasure and comfort. It is also important to consider that social withdrawal may reduce risk in the context of illicit drug use, as distancing from others can function as a protective strategy in environments that are mentally or physically unsafe. However, this same defensive pattern may become maladaptive when social withdrawal is no longer protective and may instead interfere with the formation of supportive relationships. By contrast, higher levels of love and empathy may help maintain or restore these capacities, creating a positive feedback loop that strengthens relationships and reduces the need to rely on opioids for emotional regulation. It is also possible that individuals who reported higher levels of love and empathy at baseline possessed stronger social skills than those reporting lower levels. This interpretation suggests that interventions for individuals in recovery may benefit from not only fostering prosocial emotions, but also from helping build the social skills necessary to sustain and effectively engage in experiences of love and empathy.

In the present study, after controlling for empathy and love, social support was paradoxically positively associated with increased opioid use. One interpretation is that social support in and of itself may not always be protective as not all relationships are health-promoting. Individuals embedded in social networks that lack empathy or love, or that include other drug users, may actually receive reinforcement for continued use (Latkin and Knowlton 2015; McDonald et al. 2011). This possibility complicates the prevailing narrative that “more support is always better” and highlights the need to differentiate between the quantity and quality of interpersonal relationships. These findings also suggest that healthy emotional attunement within relationships, and not the mere availability of others, may be the critical protective factor that buffers opioid use. Also, in our sample, greater anxiety was associated with more opioid use, which aligns with previous research suggesting that opioids are often used to cope with anxious arousal (Gold, Stathopoulou, and Otto 2020; Rogers et al. 2022).

Another key covariate, pain, was inversely associated with opioid use in this sample. This initially counterintuitive result may reflect withdrawal-related pain hypersensitivity: patients who use fewer opioids (as a result of addictions treatment) may experience more pain as their bodies readjust to lower levels of exogenous opioid input (Higginbotham et al. 2022). Alternatively, this finding may reflect both the stabilizing effects of methadone and the behavioral impact of pain itself. Methadone provides sustained opioid agonism, blunts withdrawal, and reduces craving, which may decrease the need for supplemental illicit opioids when pain increases. Among methadone-maintained patients, higher pain may prompt inactivity, social withdrawal, and reduced behavioral engagement, thereby suppressing drug-seeking rather than amplifying it. The moderating effects of love and empathy further clarify this pattern: for patients with lower relational resources, pain appeared to dampen motivation and engagement in drug-seeking, producing a steep decline in illicit opioid use as pain increased. In contrast, patients with higher levels of love or empathy showed a more attenuated association, suggesting that relational resources buffer how pain translates into opioid use. This finding underscores the complex and sometimes counterintuitive ways opioids interact with the body’s pain regulation systems.

Study results carry clear clinical implications as interventions that promote emotional closeness, such as compassion-based meditation techniques, emotionally-focused therapy, or structured group therapy centered on empathy-building, may offer new pathways for supporting individuals with OUD. Although the present study focuses on individuals with OUD, these findings are unlikely to be unique to opioid use alone. Prosocial emotions such as love and empathy may exert similar influences on recovery processes across other substance use disorders. Accordingly, future studies should directly examine love, empathy, and other social affiliative processes as predictors of treatment outcomes in patients with other substance use disorders. Traditional approaches to recovery that focus solely on abstinence or external accountability may benefit from explicitly targeting these prosocial capacities as mechanisms of change. In that regard, an innovative mindfulness-based intervention for OUD, Mindfulness-Oriented Recovery Enhancement (MORE), has been shown to increase feelings of universal love in people who use opioids, with consequent effects on decreasing opioid craving and opioid misuse (Garland and Jinpa 2024). Further, treatment with oxytocin (Edinoff et al. 2023; Mellentin et al. 2023) or MDMA (3,4-methylenedioxymethamphetamine) (Lee et al. 2024; Pulido-Saavedra et al. 2025) might help to improve addiction outcomes in part by stimulating feelings of love and empathy.

### Limitations

This study has several limitations. First, given its cross-sectional design, this study is unable to determine whether empathy and love are the cause, correlate, or consequence of reduced opioid use among people in recovery for OUD. Second, love and empathy were assessed via self-report measures. Although self-report measures are useful for capturing subjective experience, including how individuals perceive and experience love or empathy, they do not capture the full scope of these constructs, such as behavioral expression or reciprocity. Love and empathy are complex emotional processes that are difficult to fully assess using a single instrument. For example, the instrument we used to measure love, the Dispositional Positive Emotion Scale (DPES), measures only some aspects of this complex psychological construct. The DPES – Love items assess ease of experiencing emotional closeness, trust in others, expectations that others are dependable and supportive, and the openness to form social bonds. The DPES does not capture romantic love or feelings of affection experienced within reciprocal relationships. The measure also does not assess other important aspects of love, such as feelings of belongingness or the security and quality of close relationships. Many of these dimensions of love are likely important in addiction recovery and should be assessed in future research, as they could represent critical protective factors.

Other approaches for measuring love and empathy that capture these complexities include performance-based tasks, physiological assays (e.g., fMRI, EEG, autonomic neuroscience, biomarkers), observational methods, and ecological momentary assessment to examine how these experiences fluctuate in daily life. Use of multimethod measurement approaches can allow us to better understand the true effects of love and empathy’s role in recovery. Third, our sample consisted only of individuals with OUD who were currently in treatment, which limits generalizability. People with OUD who are not in treatment, or those receiving other forms of medication, such as buprenorphine or naltrexone, may respond differently on these assessments. Also, other unmeasured confounding variables, such as trauma history, personality traits, or cognitive functioning, could also influence how love and empathy are experienced and reported. That is, while love and empathy may function as protective factors for opioid use, this association may also be influenced by a range of confounding variables that are known to impact social bonding. For instance, interpersonal trauma and adverse childhood events often precede the development of OUD, which is known to be experienced at higher rates among individuals with OUD (Dube et al. 2003). Moreover, preexisting socio-cognitive deficits might confer risk for developing substance use (Castellanos-Ryan and Conrod 2012). An additional limitation of this study concerns the normative sample used for comparison. The normative samples were collected at different time points relative to the present OUD sample and likely differ across several demographic and contextual factors, such as socioeconomic status and clinical conditions. These normative comparisons should be interpreted as approximate benchmarks rather than definitive contrasts.

## Conclusion

This study provides novel evidence that love and empathy may function as protective factors against opioid use among people in treatment for OUD. These prosocial emotional states appear to exert effects independent of traditional clinical predictors like anxiety, depression, and pain. Given the centrality of the endogenous opioid system to both drug use and social bonding, interventions that cultivate emotionally-rich social engagement may hold promise as adjunctive treatments for OUD. Future research should continue to investigate how social emotions interact with neurobiological systems implicated in addiction, and how these pathways might be leveraged to support recovery.

## Figures and Tables

**Figure 1.**
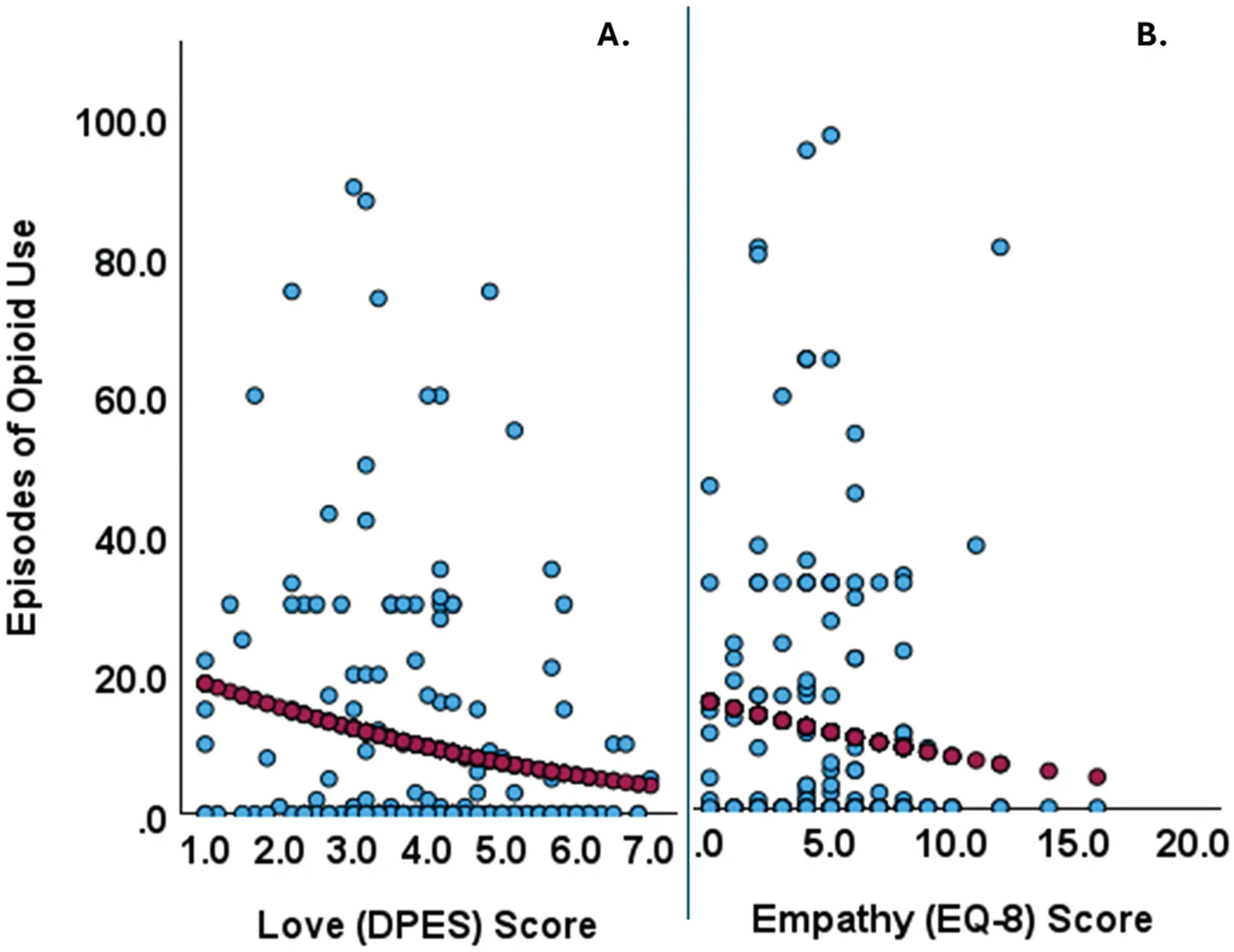
Associations among love (1A), empathy (1B), and opioid use in a sample of patients with OUD in treatment. Note: the red dotted line depicts a smooth zero-inflated Poisson (ZIP)-based mean curve plotting the association between love scores (1A), empathy scores (1B), and opioid use from zero-inflated Poisson models.

**Table 1.** Participant demographics.

Measure	Sample (*N* = 189)
**Age**	46.5 ± 13.3
Gender	
Male	100 (52.9%)
Female	87 (46.0%)
Other	2 (1.1%)
Race	
White	118 (62.4%)
Black	52 (27.5%)
Asian	3 (1.6%)
American Indian/Alaska Native	4 (2.1%)
Other	11 (5.8%)
Relationship Status	
Married	18 (9.5%)
Living with partner	30 (15.9%)
Never married	76 (40.2%)
Divorced	32 (16.9%)
Separated	10 (5.3%)
Widowed	13 (6.9%)
Not Reported	10 (5.3%)
Income	
<$10k	103 (54.5%)
$10k–24.9k	49 (25.9%)
$25k–34.9k	12 (6.3%)
$35k–49.9k	13 (6.9%)
$50k–74.9k	4 (2.1%)
≥$200k	1 (0.5%)
Prefer not to answer	7 (3.7%)
Education	
Did not complete HS	40 (21.2%)
GED or equivalent	32 (16.9%)
High school diploma	54 (28.6%)
Some college (<1 yr)	30 (15.9%)
Some college (1+ yrs), no degree	11 (5.8%)
Associate’s degree / Vocational	8 (4.2%)
Bachelor’s degree	10 (5.3%)
Graduate degree	1 (0.5%)
Other	1 (0.5%)
Prefer not to answer	2 (1.1%)
Opioid Use (past 30 days)	
Yes	77 (40.7%)
No	112 (59.3%)
Methadone Duration (years)	4.85 ± 6.03
Depression (PHQ-3)	2.45 ± 2.19
Anxiety (GAD-2)	2.59 ± 1.98
Social Support (MSPSS)	4.68 ± 1.70
Substance Use Stigma (SUSMS)	5.17 ± 2.10

Note. Values shown as Mean ± SD or n (%).

**Table 2.** Zero-inflated Poisson analysis of love and empathy as predictors of opioid use.

	Estimate	Std. Error	z Value	Significance
Count Model Coefficients				
(Intercept)	3.277	.187	17.542	<.001
Love	−.068	.021	−3.301	<.001
Empathy	−.043	.011	−4.012	<.001
Anxiety (GAD-2)	.075	.018	4.242	<.001
Depression (PHQ-3)	.015	.014	1.032	.302
Pain (PROMIS)	−.065	.016	−4.006	<.001
Social Support (MSPSS)	.122	.018	6.838	<.001
Stigma (SUSMS)	−.018	.033	−.527	.598
Zero-Inflation Model Coefficients				
(Intercept)	−.789	.972	−.812	.417
Love	.248	.138	1.797	.072
Empathy	.109	.065	1.675	.094
Anxiety (GAD-2)	−.036	.115	−.318	.751
Depression (PHQ-3)	−.044	.101	−.436	.663
Pain (PROMIS)	−.083	.095	−.878	.380
Social Support (MSPSS)	.064	.112	.576	.564
Stigma (SUSMS)	.061	.090	.676	.499

## Data Availability

Data are available from the corresponding author with a signed data access agreement.

## References

[R1] Amtmann D, Cook KF, Jensen MP, Chen W-H, Choi S, Revicki D, Cella D, Rothrock N, Keefe F, Callahan L, 2010. Development of a PROMIS item bank to measure pain interference. Pain 150 (1):173–82. doi: 10.1016/j.pain.2010.04.025. PMC2916053 20554116

[R2] Atkins DC, Baldwin SA, Zheng C, Gallop RJ, and Neighbors C. 2013. A tutorial on count regression and zero-altered count models for longitudinal substance use data. Psychology of Addictive Behaviors 27 (1):166. 10.1037/a0029508 PMC3513584 22905895

[R3] Baron-Cohen S, and Wheelwright S. 2004. The empathy quotient: An investigation of adults with asperger syndrome or high functioning autism, and normal sex differences. Journal of Autism & Developmental Disorders 34 (2):163–75. doi: 10.1023/b:jadd.0000022607.19833.00. 15162935

[R4] Burkett JP, Spiegel LL, Inoue K, Murphy AZ, and Young LJ. 2011. Activation of μ-opioid receptors in the dorsal striatum is necessary for adult social attachment in monogamous prairie voles. Neuropsychopharmacology: Official Publication of the American College of Neuropsychopharmacology 36 (11):2200–10. doi: 10.1038/npp.2011.117. PMC3176565 21734650

[R5] Castellanos-Ryan N, and Conrod P. 2012. Personality and substance misuse: Evidence for a four-factor model of vulnerability. In Drug abuse and addiction in medical illness: Causes, consequences and treatment, ed. Verster JC, Brady K, Galanter M, and Conrod P, 47–62. Springer New York. doi: 10.1007/978-1-4614-3375-0_4.

[R6] Clark L, Boileau I, and Zack M. 2019. Neuroimaging of reward mechanisms in gambling disorder: An integrative review. Molecular Psychiatry 24 (5):674–93. doi: 10.1038/s41380-018-0230-2. 30214041

[R7] Dixson DD, Anderson CL, and Keltner D. 2018. Measuring Positive emotions: An examination of the reliability and structural validity of scores on the seven Dispositional Positive emotions scales. Journal of Well-Being Assessment 2 (2–3):115–33. doi: 10.1007/s41543-019-00015-y.

[R8] Dube SR, Felitti VJ, Dong M, Chapman DP, Giles WH, and Anda RF. 2003. Childhood abuse, neglect, and household dysfunction and the risk of illicit drug use: The adverse childhood experiences study. Pediatrics 111 (3):564–72. doi: 10.1542/peds.111.3.564. 12612237

[R9] Edinoff AN, Sall S, Honore LG, Dies RM, Zaheri AR, Kataria S, Jackson ED, Shekoohi S, Cornett EM, Murnane KS, 2023. The use of oxytocin for the treatment of opioid use disorder. Current Pain and Headache Reports 27 (5):89–97. doi: 10.1007/s11916-023-01105-6. 37022563

[R10] Garland EL, and Jinpa T. 2024. Mindfulness-induced self-transcendence promotes universal love with consequent effects on opioid misuse. Behaviour Research and Therapy 175:104494. doi: 10.1016/j.brat.2024.104494. 38395015

[R11] Garnett MF, and Miniño AM. 2024. Drug overdose deaths in the United States, 2003–2023. Hyattsville, MD: National Center for Health Statistics, NCHS Data Brief, no 522. 10.15620/cdc/170565.

[R12] Gold AK, Stathopoulou G, and Otto MW. 2020. Emotion regulation and motives for illicit drug use in opioid-dependent patients. Cognitive Behaviour Therapy 49 (1):74–80. doi: 10.1080/16506073.2019.1579256. PMC6693992 30760111

[R13] Heilig M, Epstein DH, Nader MA, and Shaham Y. 2016. Time to connect: Bringing social context into addiction neuroscience. Nature Reviews Neuroscience 17 (9):592–99. doi: 10.1038/nrn.2016.67. PMC5523661 27277868

[R14] Higginbotham JA, Markovic T, Massaly N, and Morón JA. 2022. Endogenous opioid systems alterations in pain and opioid use disorder. Frontiers in Systems Neuroscience 16:1014768. doi: 10.3389/fnsys.2022.1014768. PMC9628214 36341476

[R15] Hogue A, Becker SJ, Wenzel K, Henderson CE, Bobek M, Levy S, and Fishman M. 2021. Family involvement in treatment and recovery for substance use disorders among transition-age youth: Research bedrocks and opportunities. Journal of Substance Abuse Treatment 129:108402. doi: 10.1016/j.jsat.2021.108402. PMC8380649 34080559

[R16] Inagaki TK, Ray LA, Irwin MR, Way BM, and Eisenberger NI. 2016. Opioids and social bonding: Naltrexone reduces feelings of social connection. Social Cognitive and Affective Neuroscience 11 (5):728–35. doi: 10.1093/scan/nsw006. PMC4847702 26796966

[R17] Koob GF, and Le Moal M. 1997. Drug abuse: Hedonic homeostatic dysregulation. Science 278 (5335):52–58. doi: 10.1126/science.278.5335.52. 9311926

[R18] Kroenke K, Spitzer RL, and Williams JB. 2001. The PHQ-9: Validity of a brief depression severity measure. Journal of General Internal Medicine 16 (9):606–13. doi: 10.1046/j.1525-1497.2001.016009606.x. PMC1495268 11556941

[R19] Kroenke K, Spitzer RL, Williams JB, Monahan PO, and Löwe B. 2007. Anxiety disorders in primary care: Prevalence, impairment, comorbidity, and detection. Annals of Internal Medicine 146 (5):317–25. doi: 10.7326/0003-4819-146-5-200703060-00004. 17339617

[R20] Lam J, Vuolo M, and Kelly B. 2025. Social withdrawal and prescription opioid misuse among older adults in the United States. Innovation in Aging 9 (6). doi: 10.1093/geroni/igaf052. PMC12210946 40600025

[R21] Latkin CA, and Knowlton AR. 2015. Social network assessments and interventions for health behavior change: A critical review. Behavioral Medicine 41 (3):90–97. doi: 10.1080/08964289.2015.1034645. PMC4786366 26332926

[R22] Lee YK, Gold MS, Blum K, Thanos PK, Hanna C, and Fuehrlein BS. 2024. Opioid use disorder: Current trends and potential treatments. Frontiers in Public Health 11:1274719. doi: 10.3389/fpubh.2023.1274719. PMC10850316 38332941

[R23] Loewen PJ, Lyle G, and Nachshen JS. 2009. An eight-item form of the empathy Quotient (EQ) and an application to charitable giving. Retrieved from Crcee. Umontreal *ca/Pdf/ Eight% 20Question% 20ES_final. Pdf.* http://individual.utoronto.ca/loewen/Research_files/Eight%20Question%20ES_final.pdf.

[R24] Løseth G, Eikemo M, and Leknes S. 2025. Opioid regulation of social homeostasis: Connecting loneliness to addiction. Biological Psychiatry 97 (10):971–81. doi: 10.1016/j.biopsych.2024.11.011. PMC7619192 39608698

[R25] Lyubomirsky S. 2022. Toward a New science of psychedelic social psychology: The effects of MDMA (ecstasy) on social connection. Perspectives on Psychological Science 17 (5):1234–57. doi: 10.1177/17456916211055369. 35536567

[R26] Machin AJ, and Dunbar RIM. 2011. The brain opioid theory of social attachment: A review of the evidence. Behaviour 148 (9/10):985–1025. doi: 10.1163/000579511X596624.

[R27] McDonald LJ, Griffin ML, Kolodziej ME, Fitzmaurice GM, and Weiss RD. 2011. The impact of drug use in social networks of patients with substance use and bipolar disorders. The American Journal on Addictions 20 (2):100–05. doi: 10.1111/j.1521-0391.2010.00117.x. PMC3071143 21314751

[R28] McLellan AT, Kushner H, Metzger D, Peters R, Smith I, Grissom G, Pettinati H, and Argeriou M. 1992. The fifth edition of the addiction severity index. Journal of Substance Abuse Treatment 9 (3):199–213. doi: 10.1016/0740-5472(92)90062-S. 1334156

[R29] Mellentin AI, Finn SW, Skøt L, Thaysen-Petersen D, Mistarz N, Fink-Jensen A, and Nielsen DG. 2023. The effectiveness of oxytocin for treating substance use disorders: A systematic review of randomized placebo-controlled trials. Neuroscience and Biobehavioral Reviews 151:105185. doi: 10.1016/j.neubiorev.2023.105185. 37119993

[R30] Nelson EE, and Panksepp J. 1998. Brain substrates of infant–mother attachment: Contributions of opioids, oxytocin, and norepinephrine. Neuroscience and Biobehavioral Reviews 22 (3):437–52. doi: 10.1016/S0149-7634(97)00052-3. 9579331

[R31] Panksepp J, Herman B, Conner R, Bishop P, and Scott JP. 1978. The biology of social attachments: Opiates alleviate separation distress. Biological Psychiatry 13 (5):607–18. 83167

[R32] Perlis RH, Gunning FM, Santillana M, Baum MA, Druckman JN, Ognyanova K, and Lazer D. 2025. Derivation of a 3-item patient health questionnaire as a shortened survey to capture depressive symptoms. JAMA Network Open 8 (7):e2522036–2522036. doi: 10.1001/jamanetworkopen.2025.22036. PMC12281233 40690217

[R33] Pulido-Saavedra A, Oliva HNP, Prudente TP, Kitaneh R, Nunes EJ, Fogg C, Funaro MC, Weleff J, Nia AB, and Angarita GA. 2025. Effects of psychedelics on opioid use disorder: A scoping review of preclinical studies. Cellular and Molecular Life Sciences 82 (1):49. doi: 10.1007/s00018-024-05519-2. PMC11747050 39833376

[R34] Raz S, and Berger BD. 2010. Social isolation increases morphine intake: Behavioral and psychopharmacological aspects. Behavioural Pharmacology 21 (1):39–46. doi: 10.1097/FBP.0b013e32833470bd. 19949320

[R35] Reed B, Butelman ER, and Kreek MJ. 2017. Endogenous opioid system in addiction and addiction-related behaviors. Addiction 13:196–202. doi: 10.1016/j.cobeha.2016.12.002.

[R36] Rogers AH, Garey L, Raines AM, Allan NP, Schmidt NB, and Zvolensky MJ. 2022. Anxiety sensitivity and opioid use motives among adults with chronic low back pain. Experimental and Clinical Psychopharmacology 30 (1):23. doi: 10.1037/pha0000381. 32772532

[R37] Sarnyai Z, and Kovács GL. 2014. Oxytocin in learning and addiction: From early discoveries to the present. Pharmacology Biochemistry and Behavior 119:3–9. doi: 10.1016/j.pbb.2013.11.019. 24280016

[R38] Shiota MN, Keltner D, and John OP. 2006. Positive emotion dispositions differentially associated with big five personality and attachment style. Journal of Positive Psychology 1 (2):61–71. doi: 10.1080/17439760500510833.

[R39] Simpson DD, Joe GW, Greener JM, and Rowan-Szal GA. 2000. Modeling year 1 outcomes with treatment process and post-treatment social influences. Substance Use & Misuse 35 (12–14):1911–30. doi: 10.3109/10826080009148245. 11138712

[R40] Smith LR, Earnshaw VA, Copenhaver MM, and Cunningham CO. 2016. Substance use stigma: Reliability and validity of a theory-based scale for substance-using populations. Drug & Alcohol Dependence 162:34–43. doi: 10.1016/j.drugalcdep.2016.02.019. PMC5293183 26972790

[R41] Spitzer RL, Kroenke K, Williams JBW, and Löwe B. 2006. A brief measure for assessing generalized anxiety disorder: The GAD-7. Archives of Internal Medicine 166 (10):1092–97. doi: 10.1001/archinte.166.10.1092. 16717171

[R42] Terrett G, Mercuri K, Pizarro-Campagna E, Hugrass L, Curran HV, Henry JD, and Rendell PG. 2020. Social cognition impairments in long-term opiate users in treatment. Journal of Psychopharmacology (Oxford, England) 34 (2):254–63. doi: 10.1177/0269881119875981. 31556782

[R43] Venniro M, Zhang M, Caprioli D, Hoots JK, Golden SA, Heins C, Morales M, Epstein DH, and Shaham Y. 2018. Volitional social interaction prevents drug addiction in rat models. Nature Neuroscience 21 (11):1520–29. doi: 10.1038/s41593-018-0246-6. PMC7386559 30323276

[R44] Volkow ND, Koob GF, and McLellan AT. 2016. Neurobiologic advances from the Brain disease model of addiction. The New England Journal of Medicine 374 (4):363–71. doi: 10.1056/NEJMra1511480. PMC6135257 26816013

[R45] Warren JI, Stein JA, and Grella CE. 2007. Role of social support and self-efficacy in treatment outcomes among clients with co-occurring disorders. Drug & Alcohol Dependence 89 (2–3):267–74. doi: 10.1016/j.drugalcdep.2007.01.009. PMC2753536 17329040

[R46] Yazgeç E, Bora E, İngeç S, Çıray RO, Bağcı B, and Alkın T. 2023. Social cognition in opioid use disorder. Substance Use & Misuse 58 (8):996–1003. doi: 10.1080/10826084.2023.2201845. 37096303

[R47] Zimet GD, Dahlem NW, Zimet SG, and Farley GK. 1988. The multidimensional scale of perceived social support. Journal of Personality Assessment 52 (1):30–41. doi: 10.1207/s15327752jpa5201_2. 2280326

